# Commentary: A Novel and Validated Protocol for Performing MIC Tests to Determine the Susceptibility of *Piscirickettsia salmonis* Isolates to Florfenicol and Oxytetracycline

**DOI:** 10.3389/fmicb.2018.00483

**Published:** 2018-03-14

**Authors:** Marcos Mancilla

**Affiliations:** Research and Development Laboratory, ADL Diagnostic Chile, Puerto Montt, Chile

**Keywords:** *Piscirickettsia salmonis*, minimum inhibitory concentration (MIC), antimicrobial susceptibility testing, epidemiological cut-off value, florfenicol

Contreras-Lynch et al. (2017) recently published *Piscirickettsia salmonis* epidemiological cut-off values (ECOFF) for florfenicol and oxytetracycline, and we note that the overall results of their study closely resemble those reported by us previously (Henríquez et al., 2015). The authors claim that their protocol yielded more accurate ECOFF values under the chosen experimental conditions for minimum inhibitory concentration (MIC) assessment, despite basing their findings on a considerably lower number of isolates (58 vs. 292, respectively). They also concluded that the *P. salmonis* population has experienced minor reductions in susceptibility to antibiotics. Although we are thankful for any constructive criticism regarding the precision of our study, as it enriches the discussion on this relevant research topic, we only partially agree with these statements. Indeed, we pointed out the markedly reduced susceptibility to oxytetracycline exhibited by a set of isolates analyzed in our study and warned on the potential consequences of the dissemination of such a resistant phenotype.

Notwithstanding the differences, an overlapped bimodal distribution of florfenicol susceptibility was obtained in both studies. This finding resulted intriguing because the acquisition of a resistance mechanism is generally accompanied by a notorious shift of MIC to a reduced susceptibility zone. This observation and subsequent studies that accounted for obvious phenotypic (Brevik Ø et al., 2016) and genotypic (Bravo and Martinez, 2016) differences among *P. salmonis* isolates, encouraged us to further investigate the topic considering the epidemiology of the disease. In fact, we later demonstrated that piscirickettsiosis is caused by two strains showing a degree of consistent phenotypic diversity that may justify the distinction of two subspecies (Saavedra et al., 2016). In the mentioned study, we extended our susceptibility analyses to an even larger collection of specimens (507 isolates collected in 6 years), this time grouping the isolates according to their 16S genotypes, i.e., we differentiated between EM-90-like and LF-89-like strains. Remarkably, this approach revealed only wild-type antibiotic susceptible, host-specific isolates in the EM-90-like group.

After applying a lineage segmentation on the dataset we published in 2016, the overlapped bimodal distribution of florfenicol susceptibility became clearly unimodal for the EM-90-like isolates. According to tests on normal distribution, the latter likely included only wild-type microorganisms, but this was not the case for their LF-89-like counterparts (Figure 1). A similar “strain effect” becomes evident when re-analyzing MIC values of quinolones and oxytetracycline. Therefore, regardless of the methodology used for ECOFF calculations, it seems that the overlapped bimodal distribution of MICs can at least partially be explained by the fact that data obtained from two kinds of bacteria were exploited in our studies and that of Contreras-Lynch et al. (2017). Contreras-Lynch and colleagues argued on the taxonomic heterogeneity as a source of imprecision, assuming a high level of homogeneity in their own dataset. But both Figure 1 of this commentary as well as the results presented by Contreras-Lynch et al. (2017) show that MIC data are derived from a mixed *P. salmonis* population in either case. Consequently, ECOFF calculations will yield different results when the subspecies criterion is considered.

**Figure 1:**
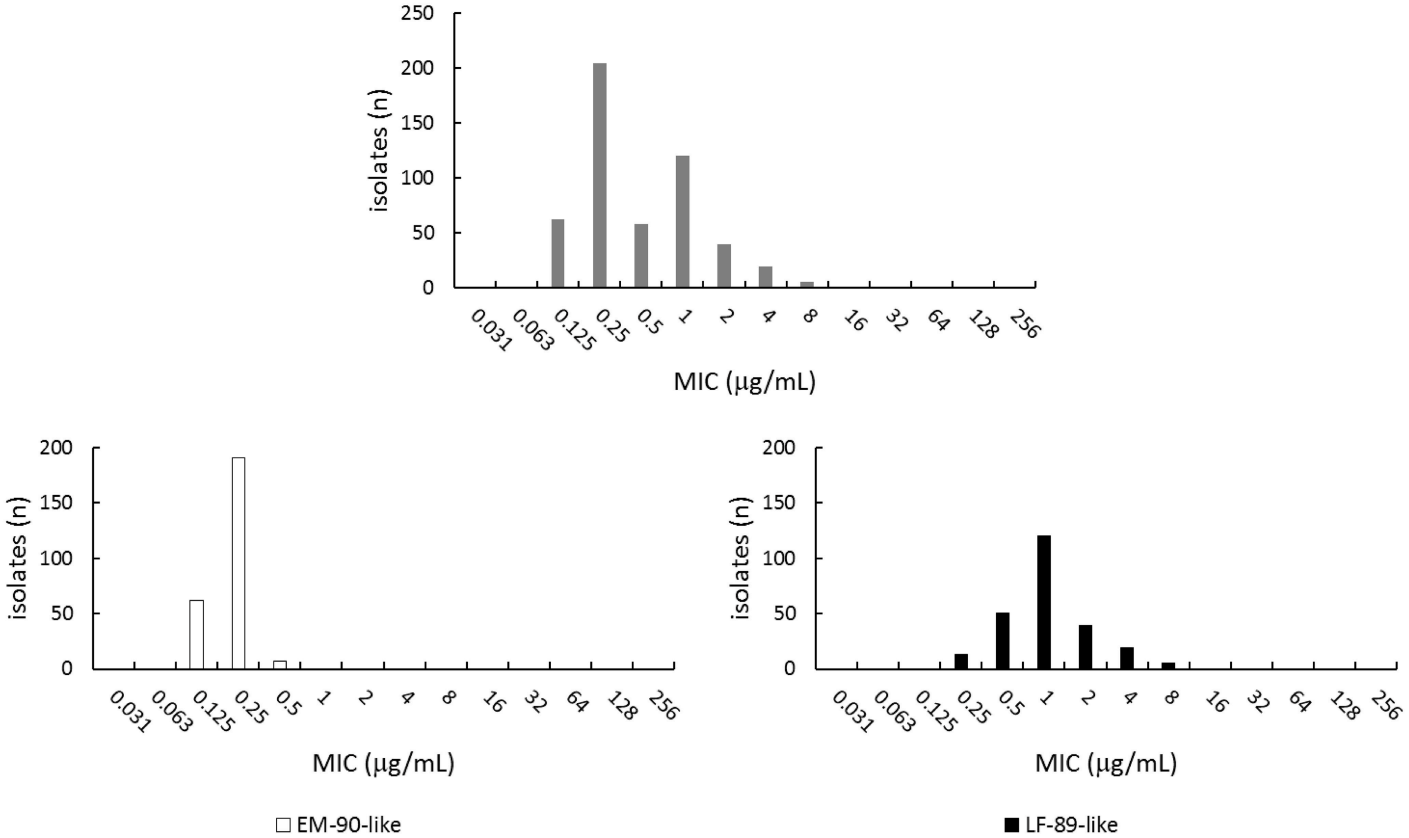
Florfenicol susceptibility profiles deduced from the dataset published in Saavedra et al. (2016). The **upper** plot shows the MIC distribution as displayed by the whole collection, which contains EM-90-like and LF-89-like *P. salmonis* isolates. The **lower** panels show strain-specific MIC distributions.

We think that the setting of ECOFF values is a starting point to establish rules for the interpretation of susceptibility to antimicrobials, yet it remains only a step on the way to clinical breakpoints. In this regard, a clinically relevant conclusion of our work is that the identification of the *P. salmonis* strain or genotype allows for a forecast of the efficacy of antimicrobial therapies, especially when a highly disseminated and fully susceptible strain is responsible for the majority of outbreaks in the current situation of piscirickettsiosis. We highlight the significance of this finding since the efforts for reducing the use of antibiotics made by the Chilean industry in recent years could benefit not only from antibiotic susceptibility surveillance, but also from genotyping of isolates. Altogether, due to the importance of *P. salmonis* genotypes as discussed above, we miss a more in-depth genetic characterization of the bacteria analyzed by Contreras-Lynch and colleagues.

## Author contributions

The author confirms being the sole contributor of this work and approved it for publication.

### Conflict of interest statement

The author declares that the research was conducted in the absence of any commercial or financial relationships that could be construed as a potential conflict of interest.
